# The Catheter Flushing Method Shows a Similar Diagnostic Yield to the Conventional Method in Brushing Cytology for Biliary Strictures

**DOI:** 10.3390/jcm13226741

**Published:** 2024-11-08

**Authors:** Sung Ill Jang, Ji Hae Nahm, See Young Lee, Seok Jeong, Tae Hoon Lee, Dong Uk Kim, Chang-Il Kwon, Jae Hee Cho, Min Je Sung

**Affiliations:** 1 Department of Internal Medicine, Gangnam Severance Hospital, Yonsei University College of Medicine, Seoul 06273, Republic of Korea; aerojsi@yuhs.ac (S.I.J.); seeyoung87@yuhs.ac (S.Y.L.); 2 Department of Pathology, Gangnam Severance Hospital, Yonsei University College of Medicine, Seoul 06273, Republic of Korea; nam2169@yuhs.ac; 3 Department of Internal Medicine, Inha University School of Medicine, Incheon 22332, Republic of Korea; inos@inha.ac.kr; 4 Department of Internal Medicine, Soonchunhyang University College of Medicine, Cheonan Hospital, Cheonan 31151, Republic of Korea; taewoolee9@gmail.com; 5 Department of Internal Medicine, CHA Gumi Medical Center, CHA University School of Medicine, Gumi 39295, Republic of Korea; amlm3@hanmail.net; 6 Digestive Disease Center, CHA Bundang Medical Center, CHA University School of Medicine, Seongnam 13496, Republic of Korea; endoscopy@cha.ac.kr

**Keywords:** catheter flushing method, brush cytology, ERCP, indeterminate biliary stricture

## Abstract

**Background/Objectives**: Endobiliary brushing is usually performed in the diagnosis of indeterminate biliary strictures; however, in this setting, brush cytology is limited by a low diagnostic yield and sensitivity. Here, we compared the catheter flushing method (CFM) with the conventional cytologic method (CCM) in terms of cellularity and diagnostic performance. **Methods**: Endobiliary brushings were obtained during endoscopic retrograde cholangiopancreatography (ERCP) from patients with biliary strictures enrolled at six tertiary hospitals. Additionally, the CFM was performed after brushing. Using liquid-based cytologic preparations of samples, we assessed the diagnostic performance of the CCM using Pap staining and the CFM using methionyl-transfer RNA synthetase 1 (MARS1) immunofluorescence staining. **Results**: From a total of 399 patients (malignant, 253; benign, 146), 374 CCM samples and 361 CFM samples contained adequate cells, with no significant difference in diagnostic yield (93.7% vs. 90.5%, respectively; *p* = 0.088). The sensitivity of the CFM (90.3%) was significantly higher than that of the CCM (75.1%; *p* < 0.001), with no significant difference in accuracy between methods (81.2% vs. 82.6%, respectively; *p* = 0.608). **Conclusions**: The diagnostic yield of the CFM was comparable to that of the CCM. Additionally, the diagnostic performance of the CFM was comparable to that of the CCM. These findings indicate that the CFM could be an additional brush cytology method for sample collection in patients with indeterminate biliary strictures. Incorporating both the CCM and CFM might be expected to improve the diagnostic yield of brush cytology in the biliary strictures. Further prospective comparative studies between the CCM and CFM using the same staining method are needed to validate these findings.

## 1. Introduction

Biliary strictures can occur in the biliary tract due to a variety of etiologies, ranging from benign conditions to malignancies [1]. While most biliary strictures are malignant, largely due to cholangiocarcinoma and pancreatic adenocarcinoma, up to 30% are benign. These benign strictures may result from conditions such as primary sclerosing cholangitis (PSC), IgG4-related sclerosing cholangitis, iatrogenic bile duct injury, and cholelithiasis. Currently, even with preoperative evaluation, up to 20% of biliary strictures remain indeterminate [2]. Despite the availability of various imaging modalities such as abdominal ultrasound, endoscopic ultrasound, computed tomography (CT), and magnetic resonance imaging, it is often difficult to determine the etiology of biliary strictures using imaging alone. Thus, pathologic confirmation is necessary for accurately diagnosing indeterminate biliary strictures, especially with suspicion of biliary tract cancer. Brush cytology is routinely performed during endoscopic retrograde cholangiopancreatography (ERCP) to determine the malignancy of biliary strictures [3]. However, the sensitivity of endoscopic brush cytology is as low as 20–40% [4,5,6]. Some researchers propose that the low sensitivity of brush cytology is mainly due to inadequate cellular sampling, although a false-negative diagnosis can occur when there is insufficient cellularity, regardless of the sampling technique used [4,7].

To improve the diagnostic yield, various endoscopic techniques have been proposed to diagnose cancer using cytology, including bile aspiration and the examination of cells in the side flaps of biliary stents [8,9,10]. Foutch et al. were the first to describe endobiliary brushing over a guidewire [11], with later studies reporting that brush cytology is superior to the simple aspiration of bile for the diagnosis of malignant strictures [12,13]. Specifically, Foutch et al. found that the sensitivity of brush cytology (33%) was higher than that of simple aspiration from bile (6%). To further increase the cancer detection rate of brush cytology, both the dilatation of the stricture and endoscopic needle aspiration have been performed together [14,15]. However, one study reports that using a pneumatic balloon or graduated dilating catheter does not increase the detection rate of brush cytology, whereas repeat brushing increases the diagnostic yield [15]. Furthermore, although using a longer cytology brush enhances cellularity, it does not improve the cancer detection rate compared with a standard brush [16]. There has also been an attempt to sample bile duct strictures using dedicated basket grasping instead of brushing [17]. In addition, post-brushing biliary lavage fluid cytology was found to be superior to bile aspiration or brush smear [18].

Recently, multiple studies showed that flushing the catheter sheath after the brush is removed increases the yield of ERCP biliary brush cytology and thereby improves diagnostic performance [19,20,21]. Wakasa et al. found that the combination of conventional smear with brush washing and flushing the sheath tube contents increases diagnostic accuracy (78.7%) compared with conventional smear procedures alone (68.8%) [19]. Nur et al. reported that ERCP brush cytology performed together with cutting the cytology brush and flushing the catheter sheath produces a high diagnostic yield (84%) [20]. Moreover, Amog-Jones et al. found that the combination of brushing and sheath tube rinsing produced moderate-to-high cellularity in 10 out of 13 (77%) specimens [21]. However, a clinically confirmative and effective method to increase the diagnostic yield of brush cytology has not been established.

Here, we compared the diagnostic yield and performance of the catheter flushing method (CFM) versus the conventional cytologic method (CCM) during ERCP in patients with indeterminate biliary strictures.

## 2. Materials and Methods

### 2.1. Study Design

This retrospective study was conducted at six tertiary medical centers. The study protocol was approved by the institutional review boards at all six facilities (3-2020-0005). The primary outcome was diagnostic performance in terms of diagnostic yield, sensitivity, specificity, positive predictive value (PPV), negative predictive value (NPV), and accuracy. The study data were accessible to all authors, each of whom reviewed and approved the final version of this manuscript.

### 2.2. Patient Selection

The inclusion criteria were as follows: biliary stricture confirmed by imaging (CT, magnetic resonance imaging, or positron emission tomography), brush cytology, and intraductal biopsy sampling by ERCP or surgical specimens; age ≥ 19 years; and no prior procedures involving the papilla. The exclusion criteria were as follows: age ≤ 18 years, pregnancy, intellectual disability, sensitivity to contrast agent, acute cholangitis, biliary strictures because of pancreatic masses, and past pancreatobiliary surgery.

### 2.3. Diagnostic Procedures

**Figure 1:**
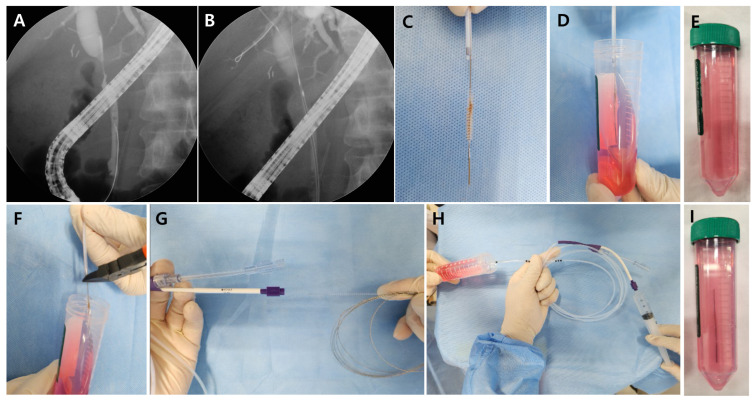
Diagnostic procedures. ERCP was used to collect bile duct brushings by making five to eight passes over the lesions (**A**,**B**). For the CCM, the brush was washed with Roswell Park Memorial Institute-1640 medium in a container (**C**–**E**). For the CFM, the brush was cut at the wire using surgical scissors into a container with preservative (**F**). After the removal of the wire, a 10 mL syringe with normal saline was used to flush residual sample from the catheter sheath into the container (**G**–**I**).

ERCP was used to collect bile duct brushings (GRBH-230-3-3.5 brush; Wilson-Cook Medical Inc., Winston-Salem, NC, USA) by making five to eight passes over the lesions (Figure 1A,B). To prepare the CCM sample, the brush was washed in a container filled with Roswell Park Memorial Institute-1640 medium (Gibco BRL, Thermo Fisher Scientific, Waltham, MA, USA) (Figure 1C–E). To prepare the CFM sample, the brush was cut at the wire using surgical scissors into a second container with the Roswell Park Memorial Institute-1640 medium (Figure 1F). After removing the wire, a 10 mL syringe with normal saline was used to flush residual sample from the catheter sheath into the second container (Figure 1G–I). After collection, samples were immediately transferred to the cytology laboratory for liquid-based ThinPrep (Cytyc Corp, Marlborough, MA, USA) slide preparation.

The containers were centrifuged, and 2–3 drops of the cell pellet were transferred to PreservCyt (Pro-Fixx; Lerner Laboratories, Pittsburgh, PA, USA). The vial containing the specimen was then placed into the ThinPrep processor, and the processing was carried out as follows. A cylinder equipped with a polycarbonate thin filter at one end was inserted into the specimen vial and rotated gently. This agitation generated a mild current that helped disperse mucus and other debris, promoting the random distribution of cells in the fluid. A vacuum was applied to the cylinder that caused most of the broken erythrocytes and debris to pass through the filter pores, while the diagnostic cells adhered to the filter’s exterior surface. The processor’s software managed cell density and adjusted the filtration rate to prevent cells from overlapping. After processing, the cylinder was removed, turned over, and pressed lightly against a positively charged slide. A slightly positive air pressure was applied to ensure the adherence of the cells to the slide. The result was a 20 mm circular smear with an even distribution of cells and minimal overlap. Finally, the slide was prepared [22,23,24,25].

After slide preparation, Pap staining was performed in the CCM for diagnostic purposes. And, to identify viable cells, MARS1 staining was conducted in the CFM. The latter was preserved (Pro-Fixx; Lerner Laboratories, Pittsburgh, PA, USA), wrapped in aluminum foil, and stored at 20 °C [26].

### 2.4. Assessment of Cellularity

Quantitative analysis of cellularity, defined as the presence of cellular material, was performed for each sample. Cellularity was evaluated by assigning a grade based on the number of epithelial cells observed on each slide [16,27,28]. Grade 0 indicates insufficient epithelial cells for interpretation (<20% of slide study area covered by epithelial cells); grade 1 indicates limited cellular material (20–50% of slide study area covered by epithelial cells); grade 2 indicates good cellularity (50–80% of slide study area covered by epithelial cells); and grade 3 indicates cellularity (>80% of slide study area covered by epithelial cells). Grades 0 and 1 were considered inadequate due to the limited amount of material available for interpretation, whereas grades 2 and 3 were considered adequate. 

### 2.5. Immunofluorescence Staining

For the methionyl-transfer RNA synthetase 1 (MARS1) immunofluorescence staining of the CFM samples, liquid-based ThinPrep slides were permeabilized in 0.3% phosphate-buffered saline containing Tween (Sigma-Aldrich, St. Louis, MO, USA) for 30 min. After two to three washes with Tris-buffered saline solution including 0.5% Tween 20 (TBS-T), slides were rinsed with distilled water and blocked for 20 min at room temperature with 3% goat serum. Slides were then incubated for 1 h at 37 °C with anti-MARS1 primary antibody (1:100; BICBIO, Suwon, Republic of Korea). After two to three washes with TBS-T, slides were incubated with secondary antibody (anti-mouse antibody Alexa Fluor 488 conjugate, 1:300; Thermo Fisher Scientific, Eugene, OR, USA) for 30 min at room temperature. The final two to three washes with TBS-T were followed by 4′,6-diamidino-2-phenylindole (Molecular Probes, Thermo Fisher Scientific) counterstaining [26,29].

### 2.6. Interpretation of Sample Staining

**Figure 2:**
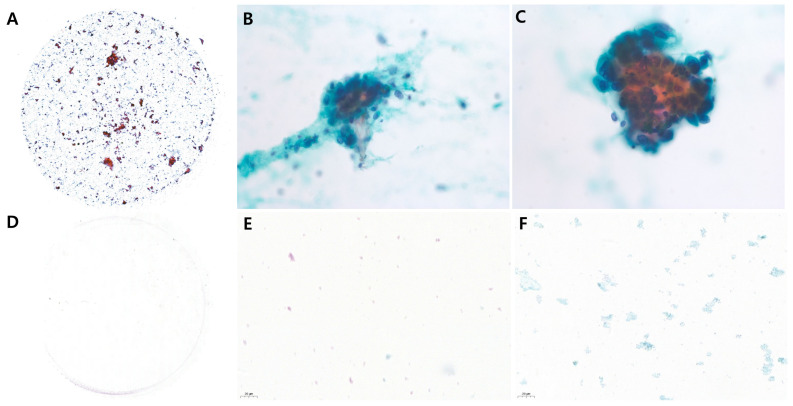
Adequate and inadequate samples in the CCM with Pap staining. Three examples of high cellularity ((**A**) original magnification; (**B**,**C**) original magnification 400×). Three examples of low cellularity ((**D**) original magnification; (**E**,**F**) original magnification 400×).

**Figure 3:**
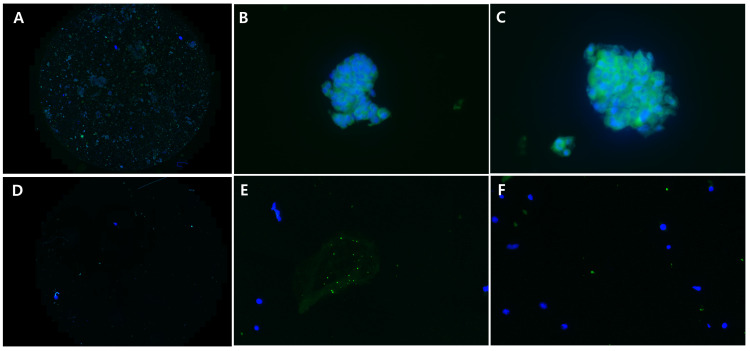
Adequate and inadequate samples in the CFM with immunofluorescence staining for MARS1. Three examples of high cellularity ((**A**) original magnification; (**B**,**C**) original magnification 400×). Three examples of low cellularity ((**D**) original magnification; (**E**,**F**) original magnification 400×).

The categories were dichotomized into adequate (cellularity grade 2 or 3) and inadequate (cellularity grade 0 or 1) groups (Figure 2 and Figure 3), and diagnostic yield was defined as the number of brush samples in the adequate group. Three pathologists (JHN, JMK, and HDC) blinded to clinical data and Papanicolaou (Pap) staining results independently examined all cellularities of the brush samples.

Brush cytologic specimens were classified into six categories—nondiagnostic, negative for malignancy, atypical, neoplastic (benign or other), suspicious for malignancy, and malignancy [30]. The ‘neoplastic (benign or other)’ category is typically assigned to cases suspected of being pancreatic neoplasms, which include pancreatic neuroendocrine tumors, solid pseudopapillary neoplasms, intraductal papillary neoplasms, and mucinous cystic neoplasms. Consequently, such cases were not included in this study. 

MARS1 immunofluorescence-stained slides were assessed in conjunction with positive (TKF-1 cells) and negative (NIH 3T3 cells) control slides. A fluorescent microscope (BX53; Olympus Corp, Tokyo, Japan) was used to view entire fields of cytologic slides at a magnification of at least 200×. Positive MARS1 staining was defined as more than one cell cluster with a high immunofluorescence signal in the cytoplasm or plasma membrane (i.e., fluorescence intensity similar to or stronger than that of positive control cells) at a magnification of at least 200×. Weak or ambiguous staining of epithelial cells was considered negative. Three pathologists blinded to clinical data and Papanicolaou (Pap) staining results independently examined all MARS1 immunofluorescence-stained cytologic tissues. Any discrepancy was resolved through the collaborative examination of the specimens [26].

### 2.7. Clinicopathologic Diagnoses

For analytic purposes, based on the CCM Pap staining, the malignant and suspicious malignancy categories were combined into a malignant subset, and the atypical and negative malignancy categories were combined into a non-malignant subset. Suspicious for malignancy is an extremely high-risk cytologic diagnostic [31]. MARS1 immunofluorescence staining was considered positive or negative, and indeterminate biliary strictures were defined as those whose etiology remained unknown after ultrasound, CT, ERCP, or cytologic assessment [32]. Brush cytology, intraductal biopsy sampling, biopsy sampling of metastatic lesions, and/or surgical specimen findings were used to determine the final clinicopathologic diagnosis. If a pathologic diagnosis could not be made, the final diagnosis relied on clinical and radiologic data collected during at least a 12-month period of follow-up. In summary, malignant biliary strictures were confirmed pathologically using brush cytology, intraductal biopsy sampling, biopsy sampling of metastatic lesions, and/or surgical specimens. Benign biliary strictures were diagnosed clinically or radiologically with at least 12 months of clinical follow-up.

### 2.8. Statistical Analysis

The diagnostic yield was analyzed using the χ^2^ test. The McNemar test was used to assess sensitivity, specificity, PPV, NPV, and accuracy, with a *p*-value < 0.05 indicating statistical significance. The Cochran–Mantel–Haenszel test and logistic regression with the generalized estimating equation (extended McNemar approach) were used to compare diagnostic performance. SPSS version 27 for Windows software (IBM Inc., Armonk, NY, USA) was used for statistical analysis.

## 3. Results

### 3.1. Patient Characteristics

In total, 399 patients with biliary strictures were included in the study. The mean patient age was 68.5 years, and there were more male than female patients (Table 1). Malignancy was detected in 253 biliary strictures (63.4%), the most common of which was cholangiocarcinoma (*n* = 214, 53.6%). The other 146 biliary strictures (36.6%) were considered benign and were most commonly considered etiology unknown (*n* = 71, 17.8%).

### 3.2. Cellularity

In a total of 399 patients, 374 samples yielded adequate cells using the CCM, while 25 samples contained inadequate cells. Additionally, 361 samples yielded adequate cells using the CFM, while 38 samples contained inadequate cells. Among the 253 patients with malignant biliary strictures, 249 samples yielded adequate cells using the CCM, while 4 samples contained inadequate cells. Furthermore, 237 samples yielded adequate cells using the CFM, while 16 samples contained inadequate cells. In the 146 patients with benign biliary strictures, 125 samples yielded adequate cells with the CCM, while 21 samples contained inadequate cells. Moreover, 124 samples yielded adequate cells with the CFM, while 22 samples contained inadequate cells. Most samples yielded adequate cells using either the CCM or CFM, with no significant difference in diagnostic yield between methods (93.7% vs. 90.5%, respectively; *p* = 0.088; Table 2). 

### 3.3. Diagnostic Performance

The CCM provided the following diagnoses: malignant (90), suspicious for malignancy (100), atypical (104), negative for malignancy (80), and nondiagnostic (25). Among the 90 malignant diagnoses made by the CCM, all were pathologically confirmed, with 85 positive and 5 negative diagnoses by the CFM. Out of the 100 brushings labeled as suspicious for malignancy, 97 were proven to be malignancies, with 89 positive and 11 negative diagnoses by the CFM. Among the 104 brushings diagnosed with atypia, 48 were confirmed to be malignancies, with 35 positive and 13 negative diagnoses by the CFM. Regarding the 80 brushings diagnosed as negative for malignancy by the CCM, 66 were found to be benign, with 14 positive and 51 negative diagnoses by the CFM. The category of nondiagnostic indicates insufficient cellularity in the conventional method with Pap staining. Negative results from the catheter flushing method also suggest inadequate cellularity. Out of the 25 brushings labeled as nondiagnostic by the CCM, 21 were determined to be benign, with 13 positive and 8 negative diagnoses by the CFM. Eight cases were classified as benign strictures—idiopathic (three cases), chronic pancreatitis with CBD stricture (three cases), choledocholithiasis (one case), and IgG4-related stricture (one case) (Table 3).

CCM and CFM results along with final clinicopathologic diagnoses are shown in Table 3. The CFM had a significantly higher sensitivity and NPV than the CCM, whereas the CCM had a significantly higher specificity and PPV than the CFM (Table 4). There was no significant difference between methods in accuracy. If both the CCM and CFM were used together, their combined sensitivity, specificity, PPV, NPV, and accuracy were 94.5%, 69.2%, 87.4%, 84.7%, and 86.7%, respectively.

## 4. Discussion

We found that the CCM and CFM had similar diagnostic yields and accuracy, whereas the CFM significantly outperformed the CCM in terms of sensitivity and NPV. Thus, the CFM could serve as an additional brush cytology technique for diagnosing indeterminate biliary strictures.

One advantage of the CFM is that it is simple and feasible. Not only did the CFM have a similar diagnostic yield as the CCM, it did not incur additional costs and only required additional flushing. Moreover, the addition of the CFM provided more cells for effectively diagnosing the etiology of indeterminate biliary strictures, as opposed to the use of the CCM alone, because the CFM additionally collected discarded cells, which consisted of those that adhered to the catheter sheath when the brush was retracted after brushing and those that remained on the brush after washing (Supplementary Figures S1 and S2). After brush cytology, the cells were bottled in one container for the CCM and two containers with the addition of the CFM. If there is no difference in the amount of adequate cells collected from the CCM and the CFM, it is possible to obtain almost twice as many cells with one brushing. As preparing two containers with one brushing has the same effect as two brushings, the addition of the CFM could save both brushing time and cost. Thus, the CFM could be beneficial to both the patient undergoing ERCP and the physician performing the procedure, as performing brush cytology multiple times during ERCP to obtain more cells not only lengthens the procedure time, but also increases the possibility of contamination.

Brush cytology typically exhibits low cellularity and yields low diagnostic results, often making additional staining challenging with the CCM alone. The CFM demonstrates cellularity comparable to that of the CCM, thereby facilitating the collection of a sufficient number of cells for analysis. Another advantage of the CFM is that obtaining a larger quantity of cells using the CFM could increase the diagnostic yield and allow further analyses, such as immunocytochemistry, immunofluorescence staining (e.g., MARS1), or fluorescence in situ hybridization. For instance, in the challenging clinical scenario of primary sclerosing cholangitis (PSC), where no mass is initially present and cytology results are inconclusive, the use of fluorescence in situ hybridization to detect polysomy is effective in identifying patients at higher risk of having or developing malignancy [33]. Moreover, next-generation sequencing (NGS) has been performed using both brush cytology and biopsy tissue [34,35], which improves the diagnosis rate of biliary tract cancer [34,36]. When compared with histological specimens, cytological samples often yield high-quality but limited nucleic acid input [35]. However, one problem with cell blocks is that the prolonged fixation in formalin can lead to C > T sequence artifacts [35]. In a study by Singhi et al., the detection rate of malignant strictures in patients with PSC was enhanced when the gene panel was evaluated using NGS in addition to the existing pathological diagnosis method through brush cytology or biopsy [34]. Furthermore, in a study by Kamp et al., the NGS mutation analysis of brush cytology in patients with PSC identified oncogenic mutations with a high level of sensitivity and specificity, demonstrating its valuable contribution as a supplementary tool [36]. To achieve more accuracy in tumor genetic analysis, such as through immunocytochemistry, immunofluorescence, and NGS, a larger number of cells from brush cytology is required. Thus, utilizing the CFM offers significant benefits by potentially reducing the need for additional ERCP procedures.

In addition, the CFM can improve the sensitivity of brush cytology. In this study, the diagnostic yield of the combined CCM and CFM was 94.7%, higher than that reported in previous studies using sheath or tube flushing (77–84%) [19,20,21]. Given that many biliary tract cancer patients have advanced disease when they are diagnosed and have a high mortality rate, a reliable diagnostic technique is important for starting treatment quickly [37]. In addition, by increasing the sensitivity of brush cytology during ERCP, it would be less necessary to perform additional invasive examinations such as endoscopic ultrasound with fine needle biopsy, repeat ERCP, or cholangioscopy. If additional diagnostic exams are not required, the risk of associated complications (e.g., bleeding, perforation, pancreatitis) is reduced [38,39] with no additional cost.

In the present study, the sensitivity of CCM in brushing cytology was 70.4%, which was higher than reported in previous studies (40–50%) [40]. In the meta-analysis, the direct smear method was employed for brush cytology, which resulted in a relatively low sensitivity [39]. However, the recent utilization of liquid-based cytology has led to an increase in the diagnostic yield of brush cytology [41]. In this study, the sensitivity is presumed to have been higher due to the application of the liquid method. We postulate that this greater sensitivity is due to our positive classification of both malignant and suspicious cases. This type of categorization tends to increase sensitivity [31]. In conventional smears, cells are spread on a glass slide using a rapid rolling motion with the brush and are then fixed with 95% alcohol for Pap staining. This can be difficult in some cases, as evenly spreading the cells on a slide requires quick movement. However, the washing, cutting, and flushing steps of the CFM are not laborious. Furthermore, compared with conventional smears, the CFM, which involves liquid-based cytology (LBC), could improve diagnostic sensitivity and accuracy [41]. Indeed, the development of LBC was driven by the need to overcome the limitations of conventional smears, including the challenges of cell clouding and blood contamination [42]. Several researchers highlight that conventional smear techniques often lead to misdiagnoses due to issues such as inadequate cellularity, artifacts caused by air drying, obscuring material, and excessively thick smears. LBC addresses these limitations by employing collection tubes, preservative fluid, and a semi-automated transfer technique [41]. Lee et al. demonstrated that LBC has a higher sensitivity and accuracy than conventional smears when brush cytology was performed with ERCP. The diagnostic performance of LBC is also comparable to that of forceps biopsies [41]. Similarly, Chun et al. showed that LBC is equally as effective as conventional smears in diagnosing solid pancreatic masses during endoscopic ultrasound-guided fine needle aspiration. In this previous study, there were no significant differences in cytomorphologic characteristics between the CFM and CCM, and the reduced presence of blood in LBC samples resulted in better visibility [43].

This study has several limitations. First, it had a retrospective design. As the two brush cytology methods employed different staining techniques, this may have affected their diagnostic performance. Differences between ThinPrep and SurePath methods of LBC may also affect diagnostic performance [44]. Nevertheless, the significant difference in sensitivity between the CCM and CFM indicates that the CFM may be a helpful additional method of brush cytology. It is also encouraging that there was no significant difference in the accuracy of the two methods. Second, MARS1 immunofluorescence staining is not yet a standard method of LBC, and its reliability may be questionable. However, as previously demonstrated, MARS1 immunofluorescence staining shows similar results to conventional Pap staining [26]. Also, due to the different staining methods, this study was unable to compare whether the combination of the CCM and CFM improves diagnostic yield compared to using the CCM or CFM alone. Future randomized controlled trials (RCTs) are necessary to evaluate the potential enhancement in diagnostic yield between the standalone methods and their combination when utilizing the same Pap stain for both the CCM and CFM. Moreover, MARS1 in the CFM serves as an auxiliary staining method to identify viable cells. Therefore, MARS1 staining in the CFM could be substituted with Pap staining. In addition, LBC has been shown to have a better diagnostic performance than conventional smear and biopsy methods [41]. Also, retrospectively, it was determined that the number of brush passes per case varied between 5 and 8. This variability in cellularity, influenced by the frequency of passes, is thought to have affected the study’s results. A prospective study is necessary to further refine and elucidate this variable. Despite these limitations, the present study demonstrates that the CFM can help increase the sensitivity of diagnosing indeterminate biliary strictures.

## 5. Conclusions

In conclusion, the diagnostic yield of the CFM was comparable to that of the CCM. This suggests that the CFM could be utilized as an additional brush cytology method in sample collection. The integration of both the CCM and CFM might be expected to improve the diagnostic yield of brush cytology in the biliary strictures. Further prospective comparative studies between the CCM and CFM using the same staining method are needed to validate these findings.

## Figures and Tables

**Table 1:** Characteristics of study participants (*n* = 399).

Variable	Value
Mean age, y (range)	68.5 (24–98)
Male-to-female ratio	243:156
Final diagnosis	
Malignant	253 (63.4)
Cholangiocarcinoma	214 (53.6)
Gallbladder adenocarcinoma	12 (3.0)
Ampullary adenocarcinoma	18 (4.5)
Neuroendocrine tumor	2 (0.5)
Other cancer (pancreatic, colon, lung)	7 (1.8)
Benign	146 (36.6)
Choledocholithiasis	52 (13.0)
Chronic pancreatitis	13 (3.3)
Postsurgical	1 (0.3)
IgG-related stricture	6 (1.5)
Primary sclerosing cholangitis	3 (0.8)
Etiology unknown	71 (17.8)
Location of biliary stricture	
Hilum	39 (9.8)
Mid	72 (18.0)
Distal	288 (72.2)
Follow up period *, months (median, range)	39.1 (12.2–87.4)

Values are *n* (%) unless otherwise defined. * Analysis for participants with benign biliary strictures.

**Table 2:** Final diagnosis and cellularity.

Diagnostic Yield According to Final Clinicopathologic Diagnosis, *n* (%)	CCM with Pap Staining		CFM with MARS1 Immunostaining		CCM + CFM		*p*-Value *
	Adequate	*p*-Value	Adequate	*p*-Value	Adequate	*p*-Value	
Malignant (*n* = 253)	249 (98.4%)	0.686	237 (93.7%)	<0.001	251 (99.2%)	reference	0.006
Benign (*n* = 146)	125 (85.6%)	0.734	124 (84.9%)	0.613	127 (86.9%)	reference	0.869
Total (*n* = 399)	374 (93.7%)	0.543	361 (90.5%)	0.021	378 (94.7%)	reference	0.088

CCM—conventional cytologic method; CFM—catheter flushing method; MARS1—methionyl-tRNA synthetase 1. * *p*-value for CCM vs. CFM.

**Table 3:** Association between brushing cytology results and clinicopathologic diagnosis.

CCM with Pap Staining	Final Clinicopathologic Diagnosis	CFM with MARS1 Immunostaining	
		Positive	Negative
Malignancy (*n* = 90)	Malignant (*n* = 90)	85	5
	Benign (*n* = 0)	0	0
Suspicious of malignancy (*n* = 100)	Malignant (*n* = 97)	86	11
	Benign (*n* = 3)	3	0
Atypical (*n* = 104)	Malignant (*n* = 48)	35	13
	Benign (*n* = 56)	15	41
Negative for malignancy (*n* = 80)	Malignant (*n* = 14)	8	6
	Benign (*n* = 66)	14	51
Nondiagnostic (*n* = 25)	Malignant (*n* = 4)	0	4
	Benign (*n* = 21)	13	8
Total (*n* = 399)	Malignant (*n* = 253)	214	39
	Benign (*n* = 146)	45	100

CCM—conventional cytology method; CFM—catheter flushing method.

**Table 4:** Diagnostic performance of the CCM and CFM.

	Sensitivity (%)	Specificity (%)	PPV (%)	NPV (%)	Accuracy (%)
CCM with Pap staining	75.1 (69.2–80.3)	97.6 (93.1–99.5)	98.4 (95.3–99.5)	66.3 (61.3–71.0)	82.6 (78.4–86.3)
CFM with MARS1 immunofluorescence staining	90.3 (85.8–93.7)	63.7 (54.6–72.2)	82.6 (79.0–85.8)	77.5 (69.5–83.8)	81.2 (76.7–85.1)
CCM + CFM	94.5 (90.7–97.0)	69.2 (59.4–77.9)	87.4 (83.8–90.3)	84.7 (76.3–90.5)	86.7 (82.6–90.2)
*p*-value	<0.001	<0.001	<0.001	0.049	0.608

Values are % (95% confidence interval). CCM—conventional cytology method; CFM—catheter flushing method; PPV—positive predictive value; MARS1—methionyl-tRNA synthetase 1; NPV—negative predictive value. *p*-value for CCM vs. CFM.

## Data Availability

The datasets generated and analyzed in the present study are available upon reasonable request to the corresponding author.

## Supplementary Materials

The following supporting information can be downloaded at: https://www.mdpi.com/article/10.3390/jcm13226741/s1, Figure S1: Evaluation of the brush before and after performing brushing on the biliary stricture. (A) Brush inside the catheter before performing brushing on the biliary stricture; (B) brush outside the catheter before performing brushing on the biliary stricture; (C) brush inside the catheter after performing brushing on the biliary stricture; (D) brush outside the catheter after performing brushing on the biliary stricture. When comparing the black arrows in Figure 1A,C, the cells on the brush were observed to adhere to the catheter sheath (black arrow in Figure 1C). When comparing the red arrows in Figure 1B,D, the red arrow in Figure 1D indicated the cells remaining on the catheter sheath. Figure S2: Evaluation of the brush after performing brushing on the biliary stricture and washing in the medium. Despite washing the brush in the medium, the cells were observed to remain on the brush (red arrows).
