# Supplementary material for: The Catheter Flushing Method Shows a Similar Diagnostic Yield to the Conventional Method in Brushing Cytology for Biliary Strictures
Source: J Clin Med. 2024 Nov 8;13(22):6741. doi: 10.3390/jcm13226741 (PMC11594799; doi:10.3390/jcm13226741)
Supplement: Supplementary file 1 [file jcm-13-06741-s001.zip › jcm-3284927-supplementary.pdf]

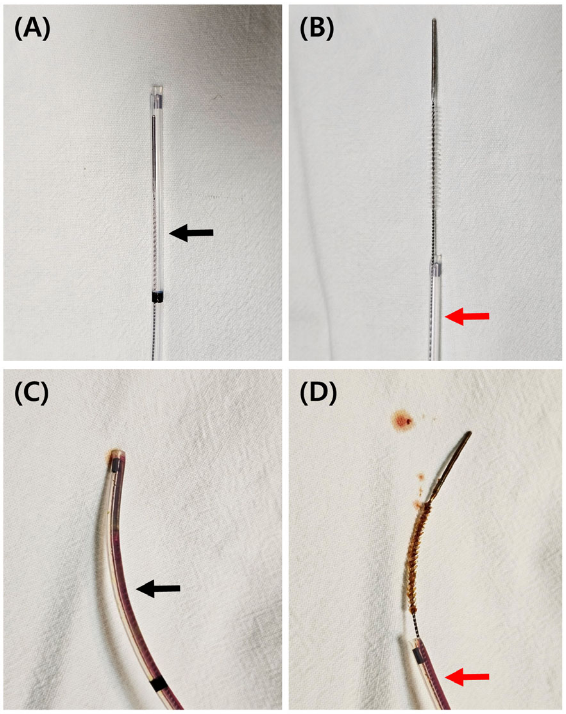

Supplementary Figure S1. Evaluation of the brush before and after performing brushing on the biliary stricture. (A) Brush inside the catheter before performing brushing on the biliary stricture, (B) Brush outside the catheter before performing brushing on the biliary stricture, (C) Brush inside the catheter after performing brushing on the biliary stricture, (D) Brush outside the catheter after performing brushing on the biliary stricture. When comparing the black arrows in Figures 1A and 1C, the cells on the brush were observed to adhere to the catheter sheath (Black arrow in Figure 1C). When comparing the red arrows in Figures 1B and 1D, the red arrow in Figure 1D indicated the cells remaining on the catheter sheath.

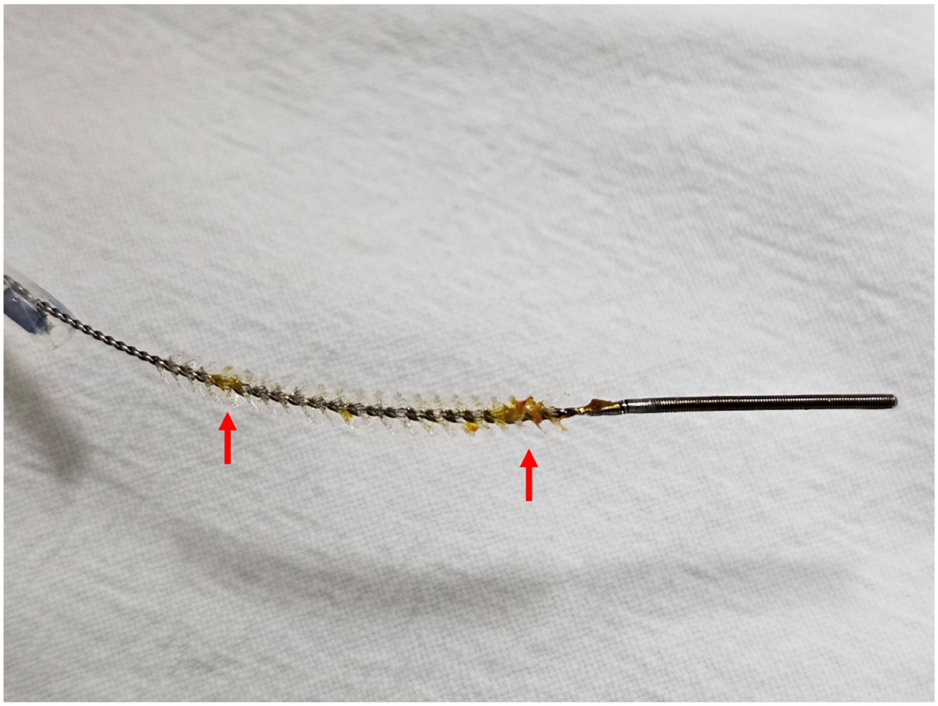

Supplementary Figure S2. Evaluation of the brush after performing brushing on the biliary stricture and washing in the medium. Despite washing the brush in the medium, the cells were observed to remain on the brush (Red arrows).
